# Hydrogen Peroxide Activated by Biochar-Supported Sulfidated Nano Zerovalent Iron for Removal of Sulfamethazine: Response Surface Method Approach

**DOI:** 10.3390/ijerph19169923

**Published:** 2022-08-11

**Authors:** Tiao Zhang, Cui Hu, Qian Li, Chuxin Chen, Jianhui Hu, Xiaoyu Xiao, Mi Li, Xiaoming Zou, Liangliang Huang

**Affiliations:** 1 College of Environmental Science and Engineering, Guilin University of Technology, Guilin 541004, China; 2 Key Laboratory of Agricultural Environmental Pollution Prevention and Control in Red Soil Hilly Region of Jiangxi Province, School of Life Science, Jinggangshan University, Ji’an 343009, China; 3 Zhongke-Ji’an Institute for Eco-Environmental Sciences, Ji’an 343016, China

**Keywords:** response surface optimization, biochar-supported sulfide-modified nanoscale zerovalent iron, sulfamethazine, Box–Behnken design

## Abstract

Biochar (BC)-supported sulfide-modified nanoscale zerovalent iron (S-nZVI/BC) was prepared using the liquid-phase reduction method for the application of the removal of sulfamethazine (SMZ) from water. The reaction conditions were optimized by the Box–Behnken response surface method (RSM). A model was constructed based on the influence factors of the removal rate, i.e., the carbon-to-iron ratio (C/Fe), iron-sulfur ratio (Fe/S), pH, and hydrogen peroxide (H_2_O_2_) concentration, and the influence of each factor on the removal efficiency was investigated. The optimal removal process parameters were determined based on theoretical and experimental results. The results showed that the removal efficiency was significantly affected by the C/Fe ratio and pH (*p* < 0.0001) but relatively weakly affected by the Fe/S ratio (*p* = 0.0973) and H_2_O_2_ concentration (*p* = 0.022). The optimal removal process parameters were as follows: 0.1 mol/L H_2_O_2_, a pH of 3.18, a C/Fe ratio of 0.411, and a Fe/S ratio of 59.75. The removal rate of SMZ by S-nZVI/BC was 100% under these conditions. Therefore, it is feasible to use the Box–Behnken RSM to optimize the removal of emerging pollutants in water bodies by S-nZVI/BC.

## 1. Introduction

Sulfonamide antibiotics (SAs) are widely used in medicine, animal husbandry, and aquaculture due to stable chemical properties, facile production, and low prices [1,2,3]. These antibiotics are far from completely absorbed by organisms during use. This incomplete absorption makes it very likely that antibiotics reaching the environment through feces and wastewater will result in toxic hazards to the environment, as well as giving rise to antibiotic-resistant bacteria (ARB) and antibiotic resistance genes (ARGs) [4] that pose a potential threat to human health [5]. Moreover, the discharge of saline wastewater containing antibiotics can degrade water quality, so the water cannot be directly used for drinking water and industrial applications [6]. Therefore, it is extremely important to carry out studies on the degradation of SAs in water.

A large number of studies have been conducted on effective antibiotic removal. Ingerslev found that the microbial method was not very effective for degrading SAs [7]. Kosutic found a comparatively higher removal effect based on the combined use of a nanomembrane and reverse osmosis membrane, which however cannot be implemented due to membrane fouling [8]. Advanced oxidation methods (AOPs) are physicochemical processes used to degrade organic substances that have a high reaction rate, strong oxidation ability, and facile control and have therefore been extensively investigated for application to water treatment [9,10,11]. These methods involve the use of hydrogen peroxide (H_2_O_2_), which is catalyzed by various materials to produce strong oxidizing hydroxide radicals (·OH) that degrade organic matter at a high rate and with a high efficiency [12,13]. Nanoscale zerovalent iron (nZVI) has been extensively investigated as a catalyst for H_2_O_2_ [12,14]. However, nZVI particles have high surface energies and magnetic properties and therefore tend to aggregate into large (micron-sized) particles, which leads to a decrease in reactivity [15]. Researchers have found that the surface modification of nZVI or loading nZVI onto a solid substrate can alleviate this problem to some extent. Biochar-supported sulfide-modified nanoscale zerovalent iron (S-nZVI/BC) has been found to be effective for the removal of organic pollutants, such as nitrobenzene and ciprofloxacin [16,17].

In previous studies on S-nZVI/BC, the carbon-to-iron (C/Fe) and iron-to-sulfur (Fe/S) ratios were considered the main influence factors for the pollutant removal efficiency [18]. The pH and H_2_O_2_ concentration are the key influence factors in a H_2_O_2_ advanced oxidation system [19]. However, no studies have been performed on optimizing the influence factors for the removal of SAs by S-nZVI/BC-activated H_2_O_2_. The response surface method (RSM) is a powerful statistical tool for studying the interaction effects of several independent variables at different levels. This method can be used to considerably reduce the required number of experiments, enabling the interaction between selected variables to be studied more quickly and systematically [20,21]. Therefore, RSM is widely used to investigate the removal of organic pollutants. For example, Zhao et al. [22] used RSM to optimize the removal of ciprofloxacin. Jung et al. [23] used RSM to optimize the removal of tetracycline hydrochloride.

In this study, S-nZVI/BC was prepared for use as a catalyst. The surface morphology and properties of S-nZVI/BC were investigated by emission scanning electron microscopy (SEM), transmission electron microscopy (TEM), micropore analysis (BET), X-ray diffraction (XRD), and X-ray photoelectron spectroscopy (XPS). The commonly used drug sulfamethazine (SMZ) was used as a pollutant in this study, and the optimal conditions for removal were determined using RSM based on Box–Behnken design (BBD) under different conditions with S-nZVI/BC as the catalyst. The C/Fe ratio, Fe/S ratio, pH, and H_2_O_2_ concentration were considered independent variables, and the effect of the interaction among these variables on the SMZ removal efficiency was investigated in order to determine the optimal reaction conditions.

## 2. Materials and Methods

### 2.1. Reagents

Ferric chloride hexahydrate (FeCl_3_·6H_2_O) ≥ 98%), sodium borohydride (NaBH_4_, ≥98%), SMZ (≥98%), sodium dithionite (Na_2_S_2_O_4_ ≥ 98%), sodium hydroxide (NaOH), hydrochloric acid HCl), n-butanol (CH_3_ (CH_2)3_OH, 99%), acetonitrile (C_2_H_3_N, ≥99.9%), and formic acid (CH_2_O_2_ ≥ 98%) were purchased from Aladdin Reagent Co., Ltd. (Shanghai, China). All the solutions used in this study were prepared using deionized water.

### 2.2. Analytical Instruments and Methods

Scanning electron microscope (SEM) equipped with an energy dispersive X-ray spectroscopy (EDS) (Regulus8100, Hitachi Limited, Tokyo, Japan) was used to observe surface morphology and localized elemental composition of composites. An X-ray diffraction analyzer (D8ADVANCE, Bruker, Karlsruhe, Germany) was applied to investigate the crystalline structure of materials. The shape and size of the particles were monitored using a JEM2100PLUS (JEOL, Tokyo, Japan) transmission electron microscope TEM. X-ray photoelectron spectroscopy (XPS) (Escalab 250xi, Thermo Fisher, Waltham, MA, USA) was used to obtain surface iron and sulfur species of the studied composites before and after the reaction. The specific surface area was analyzed by a Brunauer–Emmett–Teller (BET) analyzer (ASAP 2020, Micromeritics, Norcross, GA, USA).

### 2.3. Preparation of Biochar

Straw was crushed, ground and placed in a corundum boat that was then placed in a tube furnace. Pyrolysis at a temperature of 600 °C for 4 h in the presence of limited oxygen yielded BC. The BC was cooled to room temperature, removed from the furnace and ground through a 100-mesh sieve. The BC was added to a 3 mol/L NaOH solution at a 1:10 (g/mL) volume ratio for modification. The reaction mixture was stirred for 2 h at room temperature and allowed to stand to clarify the supernatant. The BC was washed with pure water to a near neutral pH and dried at 100 °C to yield modified BC.

### 2.4. Preparation of S-nZVI/BC

A total of 250 mL of deionized water was added to a three-necked flask and stirred for 20 min under continuous N_2_ injection. BC and FeCl_3_·6H_2_O were weighed out in a predetermined proportion and added to the flask. The flask contents were stirred for 10 min to ensure that BC and Fe^3+^ were well mixed. Na_2_S_2_O_4_ and NaBH_4_ were weighed in a predetermined proportion and dissolved in 200 mL of deionized water. The resulting solution was added dropwise to the flask using a separatory funnel (over approximately 15 min). The resulting S-nZVI particles were gradually loaded onto the BC, and the reaction was completed in 30 min to yield S-nZVI/BC with different C/Fe and Fe/S ratios. Finally, the obtained S-nZVI/BC was suction-filtered, washed with deionized water three times, dried in a vacuum drying oven, and sealed for later use.

### 2.5. Experimental Procedure

Experiments were carried out in 1-L three-necked flasks under continuous stirring using a stirrer. SMZ solutions (10 mg/L) were prepared in deionized water at different pH values (3, 6 and 9). A total of 250 mL of an SMZ solution was added to a three-necked flask, followed by the sequential addition of H_2_O_2_ and S-nZVI/BC. H_2_O_2_ was added at concentrations of 0.01 mol/L, 0.055 mol/L, and 0.1 mol/L, and the mass of added S-nZVI/BC was 0.3334 g. A timer was started immediately after the addition of S-nZVI/BC. Then, 1-mL samples were withdrawn using a disposable syringe at regular intervals and immediately passed through a 0.22-μm filter. Next, 10 μL of n-butanol were immediately added to the filtered sample to remove residual H_2_O_2_ and hydroxyl radicals [1]. The SMZ concentration was detected using our original method. The concentration of SMZ was determined by high performance liquid chromatography (LC-20AD) of the Shimadzu Corporation (Japan) and a C18 reverse-phase column (250 mm × 4.6 mm, 5 μm) of the Shimadzu Manufacturing Institute (Japan) at 28 °C. The mobile phase was acetonitrile:0.1% formic acid solution (3:7, *v*/*v*) at a flow rate of 1.0 mL·min^−1^. SMZ was detected by 268 nm UV detector [24]. The removal efficiency was calculated using the Equation (1) given below:

(1) $$\eta \;=\;\frac{C_{0}-C}{C_{0}}\;\times \;100\%$$

where η is the SMZ removal efficiency, $C_{0}$ is the initial SMZ concentration, and C is the SMZ concentration after a prescribed removal period.

### 2.6. Response Surface Method Model

Design Expert software (version 11.0.4.0) and BBD were used. The experimental design included four factors (the initial pH (3–9), the H_2_O_2_ concentration (0.01–0.1 mol/L), the C/Fe ratio (0.2–2), and the Fe/S ratio (10–60)), with three levels (−1, 0 and +1). Table 1 shows the coding and levels of the independent response variables.

The experimental data were analyzed using an RSM program, and a second-order polynomial model was obtained [25]. The Equation (2) for the variables was

(2) $$y=\beta_{0}+\Sigma_{j=1}^{k}\beta_{j}x_{j}+\Sigma_{j=1}^{k}\beta_{jj}x_{j}^{2}+\Sigma_{j=1}^{k}\sum_{i=1}^{i<j}\beta_{ij}x_{i}x_{j}+\varepsilon$$

where *y* is the predicted SMZ removal, $x_{j}$ is the coding value of the independent variable, $\beta_{0}$ is a constant term, $\beta_{j}$ is a linear coefficient, $\beta_{ij}$ is the cross-term coefficient, $\beta_{jj}$ is the quadratic coefficient, and *ε* is the error.

## 3. Results and Discussion

### 3.1. Characterization

**Figure 1:**
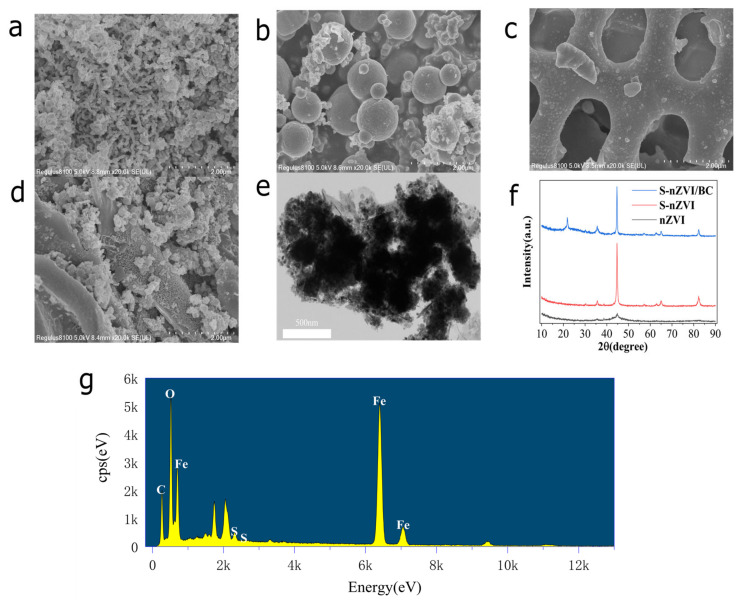
SEM images of (**a**) nZVI, (**b**) S-nZVI, (**c**) BC, and (**d**) S-nZVI/BC; (**e**) TEM images of S-nZVI/BC; (**f**) XRD spectra of nZVI, S-nZVI, and S-nZVI/BC; and (**g**) EDS map of S-nZVI/BC.

Figure 1 shows the SEM characterization results for four materials: BC, nZVI, S-nZVI, and S-nZVI/BC. nZVI has a chain structure and a relatively smooth surface (Figure 1a). S-nZVI nanoparticles are larger than nZVI particles and have rough surfaces, which may be related to S doping (Figure 1b). BC consists of a large number of flaky and porous structures (Figure 1c) that provide sufficient loading sites for S-nZVI. Figure 1d shows that S-nZVI was successfully loaded on BC. Thus, the degree of aggregation among the S-nZVI particles was considerably reduced, which increased the specific surface area of S-nZVI and the number of effective active sites for S-nZVI/BC. This result is confirmed by the TEM image of S-nZVI/BC (Figure 1e). The EDS map (Figure 1g) shows that S-nZVI/BC is mainly composed of four elements, C, O, S, and Fe, with relatively uniform distributions. XRD analysis was performed to determine the main forms of Fe and S in the materials. Figure 1f shows a distinct characteristic peak at 44.7° in all the spectra, indicating the presence of Fe^0^ [26]. The intensity of the Fe^0^ peak is significantly higher in the S-nZVI/BC spectrum than in the nZVI spectrum. A new characteristic peak at 65° indicates the production of FeS [27]. The above results revealed that S-nZVI/BC was successfully synthesized and well-dispersed on the surface of BC.

In order to further verify the synthesis of S-nZVI/BC, Brunauer–Emmett–Teller (BET) analysis was used. The results presented in Table 2 show that the specific surface area and the pore volume of nZVI were significantly reduced after vulcanization, confirming the successful vulcanization of nZVI. The results presented in Table 2 also show that BC loading resulted in a significant increase in the specific surface area and pore volume of S-nZVI, thereby increasing the number of reaction sites in S-nZVI. Compared with nZVI, the specific surface area and the total pore volume, respectively, increased by 1705.29% and 223.9%, which would create a larger contact area between S-nZVI/BC and SMZ, so as to facilitate the removal of SMZ species.

**Figure 2:**
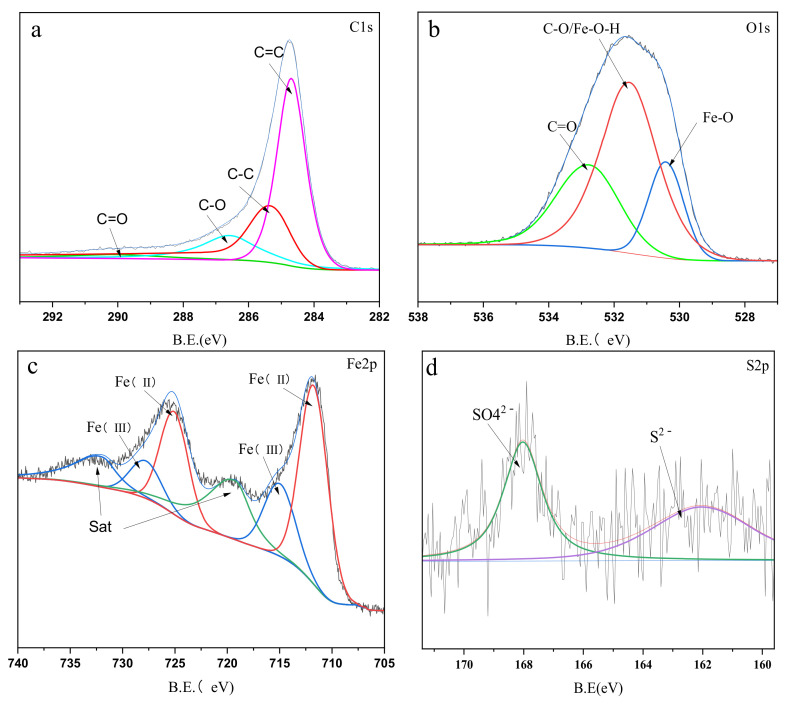
(**a**) C1s XPS spectra of S-nZVI/BC; (**b**) O1s XPS spectra of S-nZVI/BC; (**c**) Fe2p XPS spectra of S-nZVI/BC; (**d**)S2p XPS spectra of S-nZVI/BC.

The surface chemical composition of S-nZVI/BC was analyzed by XPS. Figure 2a shows that the C1s XPS spectrum could be fitted with four peaks corresponding to C=C at 284.7 eV, C–C at 285.4 eV, C-O at 286.6 eV, and C=O at 288.9 eV [28]. Figure 2b shows the O1s XPS spectrum with three characteristic peaks corresponding to Fe-O at 530.4 eV, CO/Fe-O-H at 531.4 eV, and C=O at 533.0 eV [29]. Figure 2c shows that the Fe2p XPS spectrum could be decomposed into six peaks, where the peaks at approximately 711.7 eV and 725.2 correspond to Fe (II) in FeS, and the peaks at 714.9 eV and 728.0 eV indicate the Fe (III) oxidation state [30]. The S2p spectrum exhibits two peaks at 161.83 eV and 168.3 eV (Figure 2d), corresponding to S^2−^ and SO_4_^2−^, respectively, indicating that S mainly exists in the form of FeS and that the sulfurization process is accompanied by the oxidation of sodium dithionite [31,32]. It was intuitive that the electron transfer efficiency between S-nZVI and the contaminants could be enhanced due to the excellent electrical conductivity of iron sulfides [33].

### 3.2. Model Fitting and Statistical Analysis

Experiments were performed using the parameters shown in Table 1, and the experimental results are shown in Table 3. The experimental results were analyzed using variance and significance tests, and the results are shown in Table 4. The following Equation (3) was obtained by fitting the data.

Y = +52.87 + 5.28A + 14.77B + 3.64C − 26.70D − 7.23AB − 1.26AC + 4.01AD − 7.82BC + 9.88BD − 4.53CD + 3.32A^2^ − 4.13B^2^ − 5.55C^2^ + 19.14D^2^ (3)

The F value of the regression equation model was 22.52, and the *p* value was <0.0001, indicating that the H_2_O_2_ concentration, pH, C/Fe ratio, and Fe/S ratio significantly affected SMZ removal. D^2^ had a *p* value below 0.01 and was therefore a very significant variable; BC and BD both had *p* values below 0.05 and were therefore significant variables. The pH had the most significant effect (F = 169.61) on the SMZ removal rate, and the other influence factors were, in order of significance, the C/Fe ratio, the H_2_O_2_ concentration, and the Fe/S ratio. There were interactions between the four factors, where the interaction between the initial pH and the C/Fe ratio was the most significant (F = 7.74). The correlation coefficient (R^2^) of the model was 0.9575, and the corrected correlation coefficient (R^2^ adj) was 0.915, indicating high reliability and precision for the model. In summary, the proposed model is accurate and effective for the optimization and prediction of the experimental conditions for SMZ removal by S-nZVI/BC.

**Figure 3:**
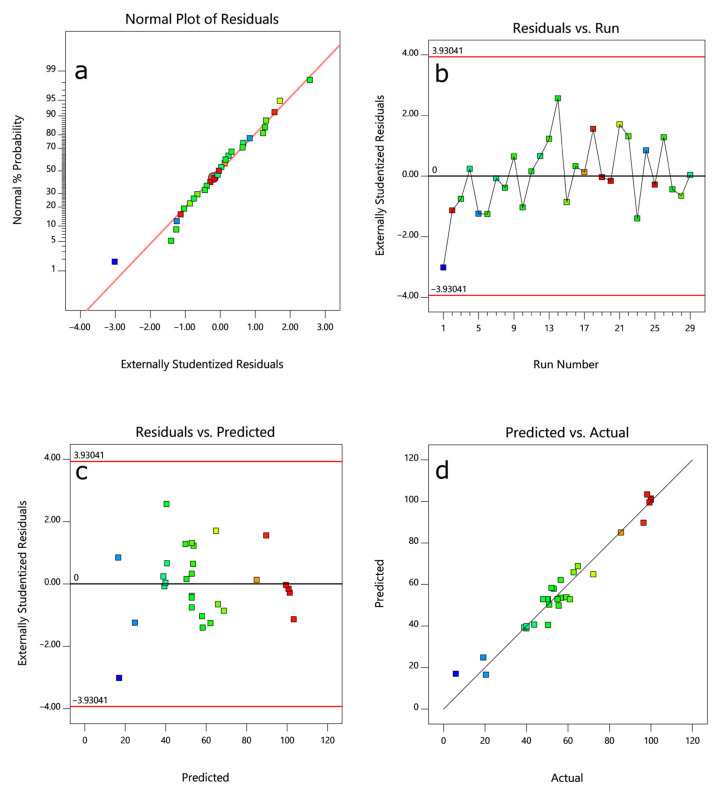
(**a**) Residual normal probability plot; (**b**) residual time plot; (**c**) residual plot based on the predicted value; (**d**) actual values versus the prediction.

Residual error analysis is performed when a model and graphical analysis tool cannot fully explain the variation in the data and can be used to analyze response surface optimization models [34]. Figure 3a verifies that the residuals obey a Gaussian distribution. The time sequence of the residuals is shown in Figure 3b. The residuals are irregularly distributed between −3.93 and 3.93 and therefore independent. The residuals were unrelated to the other variables, indicating that the model was reliable [35]. Figure 3c is the residual plot based on the predicted value. The irregular distribution of the residuals within a certain range reflects the consistency of the residuals. These three residual plots show that the quadratic polynomial model established by RSM is reliable and can accurately predict the experimental results. Figure 3d shows that the actual values are distributed relatively closely around the predicted line, confirming the statistical reliability of the predicted response surface model.

### 3.3. Analysis of Response Surfaces

The biochar provides a large specific surface area to enhance nZVI distribution equably. In S-nZVI/BC/H_2_O_2_ system degradation process, the adsorption of SMZ by biochar and nZVI was very limited through preliminary experiments. The research results of Deng et al. also proved this conclusion. [36] Thus, the essence of the S-nZVI/BC/H_2_O_2_ system degradation of SMZ is the oxidation process by •OH (Equation (4)), which obtained from H_2_O_2_ activated by BC/nZVI.

Fe^0^ + 2Fe^3+^ → 3Fe^2+^ (4)

**Figure 4:**
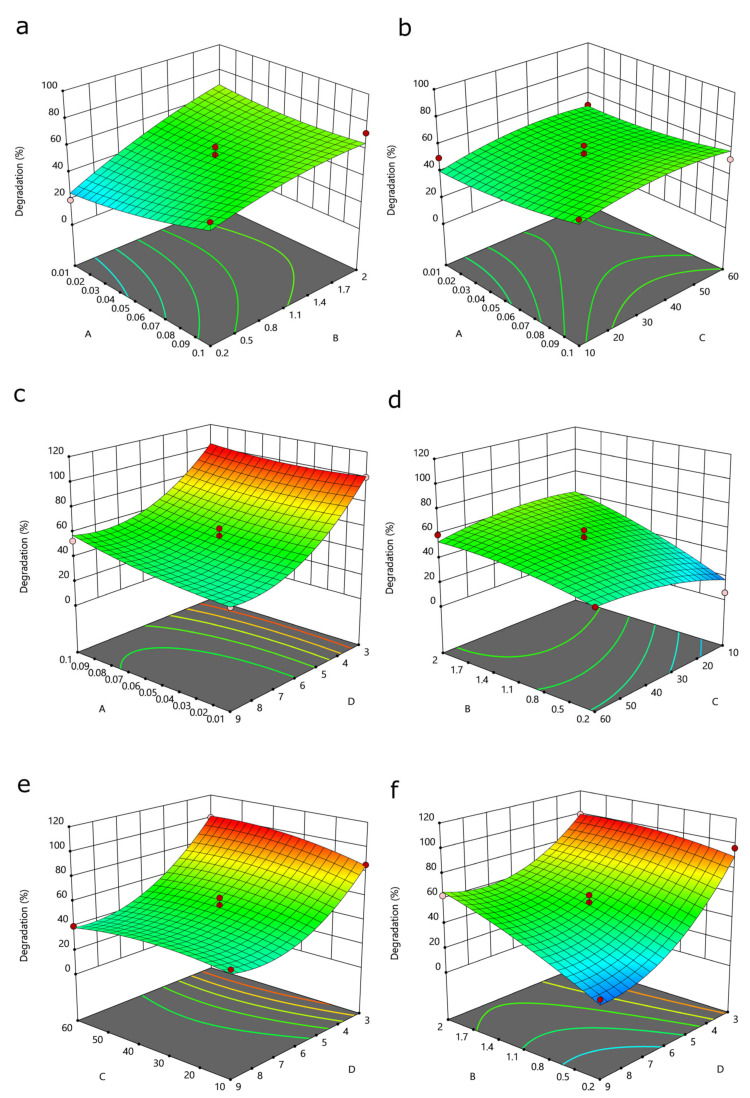
3D interaction effects between various factors: (**a**) the H_2_O_2_ concentration and C/Fe; (**b**) the H_2_O_2_ concentration and Fe/S; (**c**) the initial pH and H_2_O_2_ concentration; (**d**) C/Fe and Fe/S; (**e**) Fe/S and the initial pH; and (**f**) C/Fe and the initial pH.

Fenton oxidation involves a chain reaction between Fe^2+^ and H_2_O_2_ that catalyzes the production of a large number of free radicals to produce a strong oxidation effect [37]. The three-dimensional response surface presented in Figure 4c,e,f shows that the SMZ removal rate was highest at an initial pH of 3 and decreased as the initial pH decreased. This result may have been obtained because of the high redox potential of •OH under acidic conditions. Under acidic conditions, Fe^0^ can form Fe^2+^, which improves the reaction performance of the system (Equations (5) and (6)).

Fe^0^ + O_2_ + 2H^+^ → Fe^2+^ + H_2_O_2_ (5)

Fe^2+^ + H_2_O_2_ → Fe^3+^ + •OH + OH (6)

Acidic conditions can also slow the formation of oxide films on the surface of nanoscale zerovalent iron (nZVI), thereby reducing the loss of active sites. Under alkaline conditions, the surface of nZVI is prone to form a passivation layer, and excess OH^−^ reacts with H_2_O_2_ to form molecular oxygen (O_2_) [38]. Thus, the removal efficiency of SMZ was less than that of the acidic condition under alkaline conditions. The C/Fe ratio affects SMZ removal most significantly at pH values above 3, indicating that the adsorption of BC on SMZ is a secondary effect in SMZ removal. This result indicates that adsorption does not contribute significantly to the total reaction system. Upon increasing the H_2_O_2_ concentration, S-nZVI/BC had no significant effect on SMZ removal, perhaps because the continuous addition of H_2_O_2_ removed the •OH radicals generated in the system (Equation (7))

•OH + H_2_O_2_ → HO_2_• + H_2_O (7)

The generated hydrogen superoxide radical (HO_2_•) has a lower oxidation capacity and reactivity than •OH. Therefore, an excessive H_2_O_2_ concentration will affect the utilization rate of •OH and thereby degradation [39]. Figure 4b–e shows that the Fe/S ratio had a small effect on the SMZ removal rate. In general, the removal rate is higher when Fe/S is equal to 60. This result is the same as the research result of Dong et al. [40].

### 3.4. Model Validation

A regression model was used to determine the optimal conditions for SMZ removal by S-nZVI/BC as 0.1 mol/L H_2_O_2_, a pH of 3.18, a C/Fe ratio of 0.411, and an Fe/S ratio of 59.753. The predicted SMZ removal rate was 100% under these conditions. To verify the accuracy of this model and determine the removal under conditions that can actually be obtained in the laboratory, three parallel experiments were performed under the following conditions: 0.1 mol/L H_2_O_2_, a pH of 3, a C/Fe ratio of 0.4, an Fe/S ratio of 60, and a reaction time of 60 min. A 100% SMZ removal rate was measured under these conditions. The results of this validation experiment for the model show a high correlation between the experimental and predicted removal rates. Thus, the model used to optimize and predict the removal conditions for SMZ removal by S-nZVI/BC is accurate and reliable.

## 4. Conclusions

S-nZVI/BC was successfully synthesized and applied to the removal of SMZ. It is accurate and effective to use response surface method based on BBD to optimize and predict the experimental conditions of S-nZVI/BC removal of SMZ. Too much H_2_O_2_ will affect the utilization of hydroxyl radicals, thus affecting the degradation effect of SMZ, but Fe/S has little effect on the removal rate of SMZ, and the removal rate of SMZ is up to 100% under the best process conditions. Therefore, the model is accurate and reliable for optimizing and predicting the conditions of SMZ removal by S-nZVI/BC. From this study, it can be concluded that the use of S-nZVI/BC/H_2_O_2_ can be used successfully in the removal and reduction of SMZ and can also be optimized and controlled to maximize the removal of SMZ from aqueous solutions. It can provide valuable insight into the mechanisms of the removal of SMZ by S-nZVI/BC/H_2_O_2_ and guide future studies on sulfonamide antibiotic removal in wastewater treatment processes.

## Figures and Tables

**Table 1:** Experimental design of coding variables and actual values.

Variable	Coding	Scope and Level		
		−1	0	1
H_2_O_2_ (mol/L)	A	0.01	0.055	0.1
C/Fe	B	0.2	1.1	2
Fe/S	C	10	35	60
Initial pH	D	3	6	9

**Table 2:** Specific surface area (BET) and pore volume (TPV) of nZVI, S-nZVI and S-nZVI/BC.

	nZVI	S-nZVI	S-nZVI/BC
BET surface area (m^2^/g)	19.06	13.84	344.09
Total pore volume (cm^3^/g)	0.0806	0.0425	0.2611

**Table 3:** Group design and results of response surface experiments.

Serial Number	Variable Value				Removal Rate (%)
	A	B	C	D	
1	0	−1	−1	0	5.96
2	1	0	0	−1	98.17
3	0	0	0	0	48.00
4	0	0	1	1	40.00
5	−1	−1	0	0	19.19
6	0	1	−1	0	56.50
7	−1	0	0	1	38.98
8	0	0	0	0	50.35
9	1	0	−1	0	56.54
10	1	0	0	1	53.18
11	−1	0	1	0	50.99
12	0	0	−1	1	43.73
13	0	1	1	0	59.32
14	−1	0	−1	0	50.40
15	−1	1	0	0	64.80
16	0	0	0	0	55.00
17	0	0	−1	−1	85.60
18	0	−1	0	−1	96.48
19	0	1	0	−1	99.30
20	−1	0	0	−1	100.00
21	1	1	0	0	72.22
22	0	0	0	0	61.00
23	1	0	1	0	52.10
24	0	−1	0	1	20.44
25	0	0	1	−1	100.00
26	1	−1	0	0	55.54
27	0	0	0	0	50.00
28	0	1	0	1	62.77
29	0	−1	1	0	40.04

**Table 4:** Analysis of variance for the model for the SMZ removal rate (corresponding to the response value Y).

Source	Sum of Squares	df	Mean Square	F Value	*p* Value	
Model	15,908.66	14	1136.33	22.52	<0.0001	Significant
A	334.86	1	334.86	6.64	0.022	
B	2618.43	1	2618.43	51.9	<0.0001	
C	159.29	1	159.29	3.16	0.0973	
D	8557.35	1	8557.35	169.61	<0.0001	
AB	209.24	1	209.24	4.15	0.0611	
AC	6.33	1	6.33	0.1254	0.7286	
AD	64.24	1	64.24	1.27	0.2781	
BC	244.3	1	244.3	4.84	0.0451	
BD	390.26	1	390.26	7.74	0.0147	
CD	82.17	1	82.17	1.63	0.2226	
A^2^	71.42	1	71.42	1.42	0.2539	
B^2^	110.39	1	110.39	2.19	0.1612	
C^2^	199.65	1	199.65	3.96	0.0666	
D^2^	2375.22	1	2375.22	47.08	<0.0001	
Residual	706.33	14	50.45			
Lack of Fit	597.39	10	59.74	2.19	0.2335	Not significant
Pure Error	108.94	4	27.23			
Cor Total	16,614.99	28				
			R^2^		0.9575	
			Adjusted R^2^		0.915	
Std. Dev.	7.1		Predicted R^2^		0.7827	
Mean	58.16		Adeq Precision		16.9869	

## Data Availability

Not applicable.
